# *ACE2* and *TMPRSS2* genetic polymorphisms as potential predictors of COVID−19 severity and outcome in females

**DOI:** 10.3389/fmed.2024.1493815

**Published:** 2024-12-18

**Authors:** Sanja Matic, Dragan Milovanovic, Zeljko Mijailovic, Predrag Djurdjevic, Srdjan Stefanovic, Danijela Todorovic, Katarina Vitosevic, Vanja Canovic, Suzana Popovic, Nevena Milivojevic Dimitrijevic, Marko Zivanovic, Dragana Seklic, Sanja Aleksic, Nemanja Djordjevic, Milena Vukic, Nenad Vukovic, Nenad Filipovic, Dejan Baskic, Natasa Djordjevic

**Affiliations:** ^1^Department of Pharmacy, Faculty of Medical Sciences, University of Kragujevac, Kragujevac, Serbia; ^2^Department of Pharmacology and Toxicology, Faculty of Medical Sciences, University of Kragujevac, Kragujevac, Serbia; ^3^Department of Clinical Pharmacology, University Clinical Centre Kragujevac, Kragujevac, Serbia; ^4^Department of Infectious Diseases, Faculty of Medical Sciences, University of Kragujevac, Kragujevac, Serbia; ^5^Infectious Diseases Clinic, University Clinical Centre Kragujevac, Kragujevac, Serbia; ^6^Department of Internal Medicine, Faculty of Medical Sciences, University of Kragujevac, Kragujevac, Serbia; ^7^Clinic for Haematology, University Clinical Centre Kragujevac, Kragujevac, Serbia; ^8^Department of Genetics, Faculty of Medical Sciences, University of Kragujevac, Kragujevac, Serbia; ^9^Department of Forensic Medicine, Faculty of Medical Sciences, University of Kragujevac, Kragujevac, Serbia; ^10^Department of Forensic Medicine and Toxicology, University Clinical Center Kragujevac, Kragujevac, Serbia; ^11^Centre for Molecular Medicine and Stem Cell Research, Faculty of Medical Sciences, University of Kragujevac, Kragujevac, Serbia; ^12^Bioengineering Research and Development Center (BioIRC), Kragujevac, Serbia; ^13^Department of Sciences Institute for Information Technologies Kragujevac, University of Kragujevac, Kragujevac, Serbia; ^14^Department of Chemistry, Faculty of Sciences, University of Kragujevac, Kragujevac, Serbia; ^15^Faculty of Engineering, University of Kragujevac, Kragujevac, Serbia; ^16^Institute of Public Health Kragujevac, Kragujevac, Serbia

**Keywords:** *ACE2*, *TMPRSS2*, COVID-19, severity, outcome

## Abstract

**Introduction:**

ACE2 and TMPRSS2 represent the major gateways for SARS-CoV-2 cell entry. The presence of functional ACE2 and TMPRSS2 genetic polymorphisms that affect gene expression may affect the risk of severe form of COVID-19 and its fatal outcome.

**Material and patients:**

This observational study enrolled 178 hospitalized patients diagnosed with SARS-CoV-2 infection at the University Clinical Centre of Kragujevac, Serbia. Demographic, clinical, and laboratory data were gathered at admission. Genotyping for single nucleotide polymorphisms of *ACE2* (rs2106809 and rs2074192) and *TMPRSS2* (rs2070788 and rs4818239) was performed using the Real-Time PCR method with TaqMan assays.

**Results:**

Controlling for other factors of influence, such as CCI, N/L ratio, LDH level, and pO_2_, we showed that females with *TMPRSS2* rs2070788 A/A genotype were less likely to develop severe COVID-19 (odds ratio [OR] [95% confidence interval (95% CI)]: 0.030 [0.001; 0.862]). Additionally, the likelihood of dying of SARS-CoV-2 infection was lower in female carriers of at least one *ACE2* rs2106809 C allele (OR [95% CI]: 0.004 [0.000; 0.981]).

**Conclusion:**

Our findings indicate *TMPRSS2* rs2070788 and *ACE2* rs2106809 polymorphisms as independent predictors of severity and outcome of COVID-19 in females.

## 1 Introduction

Since the beginning of the COVID-19 pandemic, angiotensin-converting enzyme 2 (ACE2) has emerged as one of the key proteins in the pathogenesis of this infectious disease. This transmembrane protein, predominantly expressed on alveolar lung cells (1), assumes the role of a receptor for the SARS-CoV-2 virus and enables its entry into human cells (2). Variations in *ACE2* gene are known to affect the ability of ACE2 to bind to coronaviruses (3), and vast inter-ethnic differences in minor allele frequencies of its polymorphisms suggest their impact on the differences in development, course, and outcome of SARS-CoV-2 infection among populations. The possible significance of *ACE2* genotype is further supported by recent preliminary reports of an association between severe COVID-19 (4). On the other hand, the clinical effects of some of the frequent and functional *ACE2* SNPs, such as rs2106809 and rs2074192, have been associated with circulating ACE2 levels only in females (5). The observed difference in ACE2 expression in males as compared to females, which could be explained by on the X *ACE2* gene location on the X chromosome (6), is expected to contribute to sex-related disparities in COVID-19 outcomes.

The fusion of SARS-CoV-2 with the host cell and the subsequent onset of infection is facilitated by several types of proteases, the most important being transmembrane serine protease (TMPRSS2) (7). Like ACE2, TMPRSS2 is mainly expressed on the alveolar lung cells surface (8), but also in many other tissues and organs that could become the loci of complications in severe forms of COVID-19 (9). The role of TMPRSS2 in COVID-19 is further confirmed by the fact that TMPRSS2 inhibitors approved for clinical use (such as camostat mesylate—a drug used in the treatment of chronic pancreatitis) can block SARS-CoV-2 virus infection (7). The *TMPRSS2* gene is polymorphic, and some of its SNPs, such as rs2070788, have already been confirmed as a significant risk factor for influenza (10). Similarly, recent *in silico* analyses have introduced the possibility that many other *TMPRSS2* polymorphisms, including rs4818239, may affect COVID-19 infection (11). Having in mind the difference between sexes in terms of COVID-19 susceptibility and outcome (12, 13), the possible role of *TMPRSS2* genotype in this disease is further supported by its androgen-dependent regulation (14).

Given that ACE2 and TMPRSS2 represent the major gateways for SARS-CoV-2 cell entry, their sex-dependent expression strongly advocates for COVID-19 studies which would take sex into account when investigating the role of *ACE2* and *TMPRSS2* polymorphism. To meet these expectations, we evaluated the association of polymorphisms of *ACE2* and *TMPRSS2* genes with the likelihood of severe COVID-19 and fatal outcome, separately in females and males.

## 2 Material and patients

### 2.1 Study population

This prospective observational study included 178 COVID-19 patients, hospitalized at the University Clinical Center Kragujevac (UKCKG), Serbia. The sample size calculations were based on the study by Abdelsattar et al. (15), where *TMPRSS2* gene polymorphism has been assessed as a potential determinate on COVID-19 infection severity. In this study, genotyping of COVID-19 patients for *TMPRSS2* rs12329760 revealed significantly higher frequency of homozygous carriers of variant allele among severe as compared to mild cases (16.4 and 2.6%, respectively). Assuming type I error rate of 0.05 and 90% power level, minimum sample size for our study was thus estimated to 89 subjects per group, i.e., to 178 in total.

In this study, patients were diagnosed with COVID-19 by analysis of pharyngeal or nasopharyngeal swabs, using either reverse-transcriptase polymerase chain reaction (RT-PCR) as a gold-standard method (16), or rapid antigen test (RAT) as an alternative. Although sensitivity of 69.86% generally precludes the routine use of RAT for diagnosis and surveillance of COVID-19 (17), due to high specificity (99.61%) i.e., low rate of false positive results (18), it was considered valid and reliable to confirm SARS-CoV-2 infection in our study participants, including those who were asymptomatic. Patients included in the study were Caucasians of Serbian nationality, 18 years of age or older; pregnancy and breastfeeding served as exclusion criteria. A signed written informed consent was obtained from all patients or their legal representatives, and all necessary demographic and clinical data were taken from the electronic medical records. The study was conducted under the ethical standards outlined in the Declaration of Helsinki and Good Clinical Practice. The local Ethics Committee of UKCKG, Serbia, approved the study protocol by decision no. 01/20–405.

### 2.2 Data collection

The data for each individual were collected from hospital medical records, and included age, sex, symptoms and signs of COVID-19, radiological imaging results, and laboratory parameters. Pre-existing medical conditions (subsequently used to calculate the Charlson Comorbidity Index, CCI), as well as prior therapy, were assessed at admission.

Study patients were classified according to the World Health Organization (WHO) guidelines into either mild/moderate, or severe/critically severe COVID-19 cases, with the latter distinguished from the former by compromised respiratory function. All participants were followed up until hospital discharge or in-hospital death, with severity classification performed based on the worst clinical condition observed during hospitalization. Blood samples used for DNA extraction and genotyping was obtained during routine laboratory analyses.

### 2.3 DNA extraction and SNPs genotyping

Genomic DNA was extracted from 200 μL of whole EDTA blood using a commercial PureLink Genomic DNA Mini Kit (Invitrogen), according to the manufacturer’s recommendations. The quantity and quality of DNA samples were determined by spectrophotometry, using standard absorbance measurements at A260nm and A280nm on a Cary 300 UV–Vis spectrophotometer (Agilent Technologies). Samples having A260/A280 ratios ranging from 1.7 to 1.9 were suitable for genotyping (19).

Genotyping for *ACE2* and *TMPRSS2* gene polymorphisms was performed on Mic qPCR 48-well thermal cycler (BioMolecular Systems) using predesigned, commercially available TaqMan genotyping assay mix (20X; C__16098179_20 for rs2106809; C__16163821_10 for rs2074192; C___2592038_1_ for rs2070788 and C___3080270_20 for rs4818239; Applied Biosystems, Foster City, United States). TaqMan Genotyping Master Mix (2X; Applied Biosystems, Foster City, United States) was used to amplify DNA segments of interest. The final volume of the reaction mixture of 20 μL contained 10 μL TaqMan Genotyping Master Mix (2X), 0.5 μL TaqMan genotyping assay mix (20X), 0.5 μL deionized water, and 9 μL genomic DNA (or deionized water as non-template control). Two researchers independently determined the genotype calls, and repeated samples revealed no inconsistencies.

### 2.4 Statistical analyzes

Genotyping results were presented as absolute and relative frequencies of alleles and genotypes, using additive, dominant, and recessive genetic models to capture different inheritance pattern. To assess the consistency of *TMPRSS2* genotype frequencies with Mendelian inheritance, testing for the deviations from Hardy–Weinberg equilibrium (HWE) was performed by the chi-square (χ^2^) test with one degree of freedom (df = 1). Due to its location on the X chromosome, *ACE2* was tested for HWE using χ^2^—maximum-likelihood test (χ^2^-ML) with df = 1, which compares the observed with the expected genotype frequencies, where latter is calculated based on both male and female data (20). Testing for linkage disequilibrium (LD) between polymorphisms of the same gene was conducted using Haploview software, version 4.2 (Broad Institute, Cambridge, MA, United States), providing insight into non-random associations between loci.

Data were analyzed using the statistical program SPSS version 26 (IBM, Armonk, NY, United States). The association between independent variables and the risk of severe disease development or in-hospital death was first tested by univariable logistic regression; this analysis was used to evaluate the individual effect of each of the independent variables, and to select those deemed relevant to undergo subsequent analyses. To further assess the simultaneous effect of multiple factors on the outcomes of interest, the stepwise backward multivariable logistic regression was performed, with an aim to indentify predictors to be included in the statistical model that will provide best prediction of a probability of COVID-19 progression or death. To determine the strength of the observed association, odds ratios (OR) with corresponding 95% confidence intervals (CI) were calculated for each independent variable. Hosmer-Lemeshow goodness-of-fit test was used to assess the prediction model’s quality. The significance level (p) for all tests was set at less than 0.05.

## 3 Results

Genotyping of study participants for *TMPRSS2* and *ACE2* polymorphisms resulted in minor allele frequencies of 45.8, 46.9, 17.6, and 42.7% for rs2070788, rs4818239, rs2106809, and rs207419, respectively. Corresponding frequency distributions of alleles and genotype groups were separated according to sex, and presented in Supplementary Tables 1, 2. TMPRSS2 genotype frequencies were consistent with HWE for both rs2070788 (*χ^2^*(1) = 0.043, *p* = 0.836) and rs4818239 (*χ^2^*(1) = 0.303, *p* = 0.582). Similar was observed for *ACE2* rs2074192 (*χ^2^*(1) = 2.705, *p* = 0.05), but not for rs2106809, which showed statistically significant difference between expected and observed genotype frequencies (*χ^2^*(1) = 9.255, *p* = 0.01). Testing for LD between polymorphisms at *ACE2* and *TMPRSS2* loci (Supplementary Figure 1) revealed very low and moderate linkage disequilibrium between rs2106809 and rs2074192, and between rs2070788 and rs4818239, respectively.

Tables 1–4 present the frequency distribution of allele and genotype groups of *ACE2* and *TMPRSS2* polymorphisms, stratified by sex, according to the severity of SARS-CoV-2 infection and its outcome. Univariate logistic regression did not detect any significant association of *ACE2* alleles and genotype groups with either severity (Table 1) or outcome (Table 3) of COVID-19. Contrarily, *TMPRSS2* rs2070788 A and rs4818239 C alleles were significantly associated with a two- to three-fold decrease in the likelihood of developing severe COVID-19 in both females and males (Table 2), while the odds for in-hospital death decreased in the presence of rs2070788 A and rs4818239 C in females and males, respectively (Table 4).

**Table 1:** *ACE2* frequency of allele and genotype group in male and female according severity of SARS-CoV-2 infection.

		Male				Female			
		Mild disease; *n* = 52	Severe disease; *n* = 54	OR [95%CI]^a^	*p*	Mild disease; *n* = 39	Severe disease; *n* = 33	OR [95%CI]^a^	*p*
Allele									
rs2106809	T	73.1(38)	77.8(42)	1	*ref.*	89.7(70)	84.8(56)	1	*ref.*
	C	26.9(14)	22.2(12)	0.776 [0.319; 1.883]	*0.978*	10.3(8)	15.2(10)	1.563 [0.578; 4.221]	*0.379*
rs2074192	G	62.2(28)	69.2(36)	1	*ref.*	51.3(40)	51.5(34)	1	*ref.*
	A	37.8(17)	30.8(16)	0.732 [0.315; 1.700]	*0.574*	48.7(38)	48.5(32)	0.991 [0.514; 1.909]	*0.978*
Genotype group									
Additive genetic model									
rs2106809	T/T	NA				84.6(33)	72.7(24)	1	*ref.*
	T/C					10.3(4)	24.2(8)	2.75 [0.742; 10.196]	*0.130*
	C/C					5.1(2)	3(1)	0.688 [0.059; 8.026]	*0.765*
rs2074192	G/G	NA				30.8(12)	24.2(8)	1	*ref.*
	G/A					41(16)	54.5(18)	1.687 [0.551; 5.171]	*0.360*
	A/A					28.2(11)	21.2(7)	0.955 [0.259; 3.514]	*0.944*
Dominant genetic model									
rs2106809	T/T	NA				84.6(33)	72.7(24)	1	*ref.*
	T/C + C/C					15.4(6)	27.3(9)	2.062 [0.647; 6.573]	*0.221*
rs2074192	G/G	NA				30.8(12)	24.2(8)	1	*ref.*
	G/A + A/A					69.2(27)	75.8(25)	1.389 [0.487; 3.957]	*0.539*
Recessive genetic model									
rs2106809	T/T + T/C	NA				94.9(37)	97.0(32)	1	*ref.*
	C/C					5.1(2)	3.0(1)	0.578 [0.050; 6.677]	*0.661*
rs2074192	G/G + G/A	NA				71.8(28)	78.8(26)	1	*ref.*
	A/A					28.2(11)	21.2(7)	0.685 [0.231; 2.033]	*0.496*

The results are presented as relative (%) and absolute (n) frequency; p, probability; ref., reference category; NA, not applicable.

a univariable logistic regression.

**Table 2:** *TMPRSS2* frequency of allele and genotype groups in male and female according severity of SARS-CoV-2 infection.

		Male				Female			
		Mild disease; *n* = 52	Severe disease; *n* = 54	OR [95%CI]^a^	*p*	Mild disease; *n* = 39	Severe disease; *n* = 33	OR [95%CI]^a^	*p*
Allele									
rs2070788	G	36.5(38)	50.9(55)	1	*ref.*	35.9(28)	63.6(42)	1	*ref.*
	A	63.35(66)	49.1(53)	0.555 [0.32; 0.961]	***0.036***	64.1(50)	36.4(24)	0.320 [0.162; 0.633]	***0.001***
rs4818239	T	45.1(47)	62(67)	1	*ref.*	43.6(34)	62.1(41)	1	*ref.*
	C	54.8(57)	38(41)	0.505 [0.292; 0.873]	***0.014***	56.4(44)	37.9(25)	0.471 [0.241; 0.92]	***0.027***
Genotype groups									
Additive genetic model									
rs2070788	GG	25.0(13)	22.2(12)	1	*ref.*	12.8(5)	24.2(8)	1	*ref.*
	GA	53.8(28)	46.3(25)	0.967 [0.373; 2.506]	*0.945*	43.6(17)	51.5(17)	0.625 [0.17; 2.302]	*0.480*
	AA	21.2(11)	31.5(17)	1.674 [0.562; 4.986]	*0.355*	43.6(17)	24.2(8)	0.294 [0.073; 1.19]	*0.086*
rs4818239	TT	26.9(14)	27.8(15)	1	*ref.*	38.5(15)	24.2(8)	1	*ref.*
	TC	48.1(25)	50(27)	1.008 [0.406; 2.502]	*0.986*	43.6(17)	48.5(16)	1.765 [0.589; 5.283]	*0.310*
	CC	25.0(13)	22.2(12)	0.862 [0.295; 2.513]	*0.785*	17.9(7)	27.3(9)	2.411 [0.652; 8.92]	*0.187*
Dominant genetic model									
rs2070788	GG	25(13)	22.2(12)	1	*ref.*	12.8(5)	24.2(8)	1	*ref.*
	GA + AA	75(39)	77.8(42)	1.167 [0.475; 2.862]	*0.736*	87.2(34)	75.8(25)	0.460 [0.134; 1.574]	*0.216*
rs4818239	TT	26.9(14)	27.8(15)	1.	*ref.*	38.5(15)	24.2(8)	ref.	*ref.*
	TC + CC	73.1(38)	72.2(39)	0.958 [0.408; 2.251]	*0.921*	61.5(24)	5.8(25)	1.953 [0.701; 5.442]	*0.200*
Recessive genetic model									
rs2070788	GG + GA	78.8(41)	68.5(37)	1	*ref.*	56.4(22)	75.8(25)	1	*ref.*
	AA	21.2(11)	31.5(17)	1.713 [0.711; 4.125]	*0.230*	43.6(17)	24.2(8)	0.414 [0.15; 1.145]	*0.089*
rs4818239	TT + TC	80.8(42)	70.4(38)	1	*ref.*	84.6(33)	72.7(24)	1	*ref.*
	CC	19.2(10)	29.6(16)	1.768 [0.716; 4.366]	*0.216*	15.4(6)	27.3(9)	2.062 [0.647; 6.573]	*0.221*

Bold values are statistically significant. The results are presented as relative (%) and absolute (n) frequency; p, probability; ref., reference category.

a univariable logistic regression.

**Table 3:** *ACE2* frequency of allele and genotype group in male and female according outcome of SARS-CoV-2 infection.

		Male				Female			
		Hospital-discharged; *n* = 85	In-hospital death; *n* = 21	OR [95%CI]^a^	*p*	Hospital-discharged; *n* = 54	In-hospital death; *n* = 18	OR [95%CI]^a^	*p*
Allele									
rs2106809	T	72.9(62)	85.7(18)	1	*ref.*	87(94)	88.9(32)	1	*ref.*
	C	27.1(23)	14.3(3)	0.449 [0.121; 1.669]	*0.232*	13(14)	11.1(4)	0.839 [0.258; 2.735]	*0.771*
rs2074192	G	68.8(53)	55.0(11)	1	*ref.*	50.9(55)	52.8(19)	1	*ref.*
	A	31.2(24)	45.0(9)	1.807 [0.662; 4.933]	*0.248*	49.1(53)	47.2(17)	0.929 [0.436; 1.976]	*0.847*
Genotype group									
Additive genetic model									
rs2106809	T/T	NA				77.8(42)	83.5(15)	1	*ref.*
	T/C					18.5(10)	11.1(2)	0.560 [0.110; 2.854]	*0.485*
	C/C					3.7(2)	5.6(1)	1.400 [0.118; 16.581]	*0.790*
rs2074192	G/G	NA				27.8(15)	27.8(5)	1	*ref.*
	G/A					46.3(25)	50.0(9)	1.080 [0.304; 3.834]	*0.905*
	A/A					25.9(14)	22.2(4)	0.857 [0.191; 3.853]	*0.841*
Dominant genetic model									
rs2106809	T/T	NA				77.8(44)	83.3(15)	1	*ref.*
	T/C + C/C					22.2(12)	16.7(3)	0.700 [0.173; 2.827]	*0.616*
rs2074192	G/G	NA				27.8(15)	27.8(5)	1	*ref.*
	G/A + A/A					72.2(39)	72.2(13)	1.000 [0.304; 3.290]	*1.000*
Recessive genetic model									
rs2106809	T/T + T/C	NA				96.3(52)	94.4(17)	1	*ref.*
	C/C					3.7(2)	5.6(1)	1.529 [0.130; 17.939]	*0.735*
rs2074192	G/G + G/A	NA				74.1(40)	77.8(14)	1	*ref.*
	A/A					25.9(14)	22.2(4)	0.816 [0.230; 2.898]	*0.754*

The results are presented as relative (%) and absolute (n) frequency; p, probability; ref., reference category; NA, not applicable.

a univariable logistic regression.

**Table 4:** *TMPRSS2* frequency of allele and genotype groups in male and female according to outcome of SARS-CoV-2 infection.

		Male				Female			
		Hospital-discharged; *n* = 85	In-hospital death; *n* = 21	OR [95%CI]^a^	*p*	Hospital-discharged; *n* = 54	In-hospital death; *n* = 18	OR [95%CI]^a^	*p*
Allele									
rs2070788	G	43.5(74)	45.2(19)	ref.		42.6(46)	66.7(24)	ref.	
	A	56.5(96)	54.8(23)	0.933 [0.473; 1.84]	*0.842*	57.4(62)	33.3(12)	0.371 [0.168; 0.818]	***0.014***
rs4818239	T	49.4(84)	71.4(30)	ref.		50.0(54)	58.3(21)	ref.	
	C	50.6(86)	28.6(12)	0.391 [0.188; 0.814]	***0.012***	50.0(54)	41.74(15)	0.714 [0.333; 1.531]	*0.387*
Genotype groups									
Additive genetic model									
rs2070788	GG	23.5(20)	23.8(5)	ref.		13.0(7)	33.3(6)	ref.	
	GA	50.6(43)	47.6(10)	0.93 [0.281; 3.081]	*0.906*	48.1(26)	44.4(8)	0.359 [0.093; 1.382]	*0.136*
	AA	25.9(22)	28.6(6)	1.091 [0.288; 4.135]	*0.898*	38.9(21)	22.2(4)	0.222 [0.048; 1.023]	*0.054*
rs4818239	TT	28.2(24)	23.8(5)	ref.		37(20)	16.7(3)	ref.	
	TC	50.6(43)	42.9(9)	1.005 [0.302; 3.342]	*0.994*	44.4(24)	50.0(9)	2.500 [0.595; 10.500]	*0.211*
	CC	21.2(18)	33.3(7)	1.867 [0.509; 6.851]	*0.347*	18.5(10)	33.3(6)	4.000 [0.824; 19.423]	*0.086*
Dominant genetic model									
rs2070788	GG	23.5(20)	23.8(5)	ref.		13.0(7)	33.3(6)	ref.	
	GA + AA	76.5(65)	76.2(16)	0.985 [0.321; 3.025]	*0.978*	87.0(47)	66.7(12)	0.298 [0.084; 1.051]	*0.060*
rs4818239	TT	28.2(24)	23.8(5)	ref.		37.0(20)	16.7(3)	ref.	
	TC + CC	13.8(61)	76.2(16)	1.259 [0.415; 3.819]	*0.684*	63.0(34)	83.3(15)	2.941 [0.757; 11.426]	*0.119*
Recessive genetic model									
rs2070788	GG + GA	74.1(63)	71.4(15)	ref.		61.1(33)	77.8(14)	ref.	
	AA	25.9(22)	28.6(6)	1.145 [0.395; 3.319]	*0.802*	38.9(21)	22.2(4)	0.449 [0.130; 1.549]	*0.205*
rs4818239	TT + TC	76.5(65)	71.4(15)	ref.		83.3(45)	66.7(12)	ref.	
	CC	23.5(20)	28.6(6)	1.300 [0.445; 3.795]	*0.631*	16.7(9)	33.3(60)	2.50 [0.743; 8.413]	*0.139*

Bold values are statistically significant. The results are presented as relative (%) and absolute (n) frequency; p, probability; ref., reference category.

a univariable logistic regression.

Multivariable logistic models, developed to evaluate the association of genetic polymorphisms in *ACE2* and *TMPRSS2* genes, laboratory and clinical parameters at admission, and the likelihood of developing severe COVID-19 and lethal outcome in female and male patients separately (Table 5; Supplementary Table 3), revealed significance of tested polymorphisms only in female population. The final model of highest predictive quality (Hosmer-Lemeshow test: χ^2^(7) = 4.016, *p* = 0.778; Nagelkerke R^2^ = 75.2%, correctly classified 83.3% of cases) showed that females carrying both major alleles of *TMPRSS2* rs2070788 were less likely to develop severe COVID-19. On the other hand, the best fitting model for outcome prediction (Hosmer-Lemeshow test: χ^2^(7) = 0.341, *p* = 1.000; Nagelkerke R^2^ = 77.2%; adequately classified 92.4% of cases) revealed that the likelihood of surviving SARS-CoV-2 infection was 95% higher in females carrying at least one minor allele of *ACE2* rs2106809 compared to those carrying two major alleles.

**Table 5:** Summary of variable estimates from the best fitting models of multiple logistic regression analysis regarding severity and outcome of SARS-CoV-2 infection in females.

	Variables	B	S.E.	Wald	*p*	OR	[95%CI]
Severity	LDH	0.007	0.004	4.172	**0.041**	1.007	[1.000; 1.015]
	pO_2_	−1.859	0.742	6.272	**0.012**	0.156	[0.036; 0.668]
	*ACE2* rs2074192^a^	2.729	1.648	2.743	0.098	15.317	[0.606; 386.952]
	*TMPRSS2* rs2070788^b^	−3.521	1.721	4.186	**0.041**	0.030	[0.001; 0.862]
	Constant	10.687	5.486	3.795	0.051	43772.445	
Outcome	CCI	1.863	0.719	6.714	***0.010***	6.445	[1.575; 26.385]
	N/L	0.442	0.214	4.268	***0.039***	1.556	[1.023; 2.365]
	*ACE2* rs2106809^c^	−5.502	2.797	3.868	***0.049***	0.004	[0.000; 0.981]
	ACE Inhibitors^d^	2.952	1.710	2.979	0.084	19.149	[0.670; 547.169]
	Constant	−11.122	3.963	7.877	0.005	0.000	

Values in bold indicate statistically significant results. LDH, lactat dehydrogenase; pO_2_, partial pressure of oxygen; CCI, Charlson comorbidity index; N/L, neutrophil to lymphocyte ratio; B, the regression coefficient; S.E., the standard error; Wald χ^2^, Wald test statistics for the degree of freedom of 1 (df = 1); OR, odds ratio; 95% CI, the 95% confidence interval for the estimated OR; p, the probability.

a dominant model, C/C as reference category.

b recessive model, G/G + G/A as reference category.

c dominant model, A/A as reference category.

d no ACE inhibitors as reference category.

## 4 Discussion

Although the WHO announced in May 2023 that COVID-19 no longer represents a public health emergency of international concern, the increase in the frequency of new subvariants of Omicron SARS-CoV-2 continue to attract public attention (21). These new strains, especially BA.2.86 (Pirola) and JN.1, are characterized by more than 30 mutations on the spike (S) protein (22), which heighten their ability to evade vaccine-induced immunity and increase the prevalence of COVID-19 worldwide (23). Therefore, even 4 years after the initial outbreak, the clinical presentation of SARS-CoV-2 infection is still variable, ranging from completely asymptomatic, to critically severe forms that are potentially life-threatening (24).

Despite well-established risk factors like age, sex, and chronic diseases, early identification of individuals at risk for severe viral infections remains important clinical challenge. Genetic variations, particularly of genes coding for receptors and enzymes responsible for viral entrance into the human organism, can significantly influence host response to SARS-CoV-2 infection (25). Our research highlights the potential of genetic markers associated with *ACE2* and *TMPRSS2* in predicting the severity and outcome of COVID-19, especially in females. Specifically, our results indicate that female carriers of both major A alleles of *TMPRSS2* rs2070788 and at least one minor G allele of *ACE2* rs2106809 have lower odds of severe COVID-19 and fatal outcome, respectively. To our knowledge, our study is the first to report the association of *ACE2* rs2106809 with SARS-CoV-2 infection outcome, and one of the very few to describe the protective role of the *TMPRSS2* rs2070788 A/A genotype on COVID-19 severity, both in females. Additionally, our study reaffirms the significance of certain clinical factors, such as CCI, N/L ratio (26), LDH level (27), and pO_2_ at admission, in predicting COVID-19 severity and mortality.

*ACE2* gene maps to X chromosome and codes for transmembrane protein ACE2 (28). There are two known forms of human ACE2: membrane-bound (mACE2) and soluble (sACE2), latter arising from the former by ADAM17-mediated cleavage and subsequent shedding of its extracellular catalytic domain from the membrane (28, 29). In the context of SARS-CoV-2 infection, mACE2 is identified as a receptor (7) that triggers conformational change of the virion (30), fostering its TMPRSS2-mediated proteolytic cleavage and consequent entry into the target cell (31). At the same time, sACE2 competes with mACE2 for binding to SARS-CoV-2, but without prompting entrance to the cell; this reduces the number of viral particles that will attach to the cell surface, providing protective effect against SARS-CoV-2 infection (8, 31). In the context of COVID-19 severity and mortality, both mACE2 and sACE2 counteract the effects of its homolog angiotensin-converting enzyme (ACE) within renin-angiotensin-aldosterone system (RAAS) axis, decreasing the level of angiotensin (Ang) II in favor of Ang (1–7). This leads to an increased activation of MAS instead of AT1R receptor, creating vasodilatatory and anti-inflammatory effects that decrease the risk of severe form of the disease and its fatal outcome (32). Both of these cascades of events correspond well to the suggested considering COVID-19 as a dual phase phenomenon: in the first phase of infection, ACE2 promotes viral entry, but in the later phase, it acts protectively against respiratory failure and COVID-19-related death (33). However, upon infection, virion-bound mACE2 gets internalized along with the viral particle, decreasing the number of available ACE2 molecules on the cell membrane (34). Moreover, during the course of the disease, SARS coronaviruses, including SARS-CoV-2, downregulate ACE2 cell surface expression (35, 36). Within RAAS, reduction of mACE2 gives advantage to ACE, which shifts abovementioned Ang II/Ang (1–7) proportion toward the former, prompting its binding to ATR1. This further downregulates mACE2 (37), but also initiates a crucial mechanism of ADAM17 activation (38), which in turn triggers an increase in sACE2 level at the expense of mACE2 (28, 29). In line with these observations, previous studies reported elevated levels of sACE2 in COVID-19 patients that did not survive the infection, suggesting its potential role as an independent biomarker of COVID-19 mortality (39). The persistency of high sACE2 throughout hospitalization in SARS-CoV-2 non-survivors, as well as the clear disparity in its level observed between males and females, point toward the role of both *ACE2* genetic polymorphism and sex in determining the proposed prognostic value of sACE2 in COVID-19.

*ACE2* rs2106809 is an intronic variant that alters gene splicing efficiency (5, 40), with minor C allele being associated with increased *ACE2* expression in multiple tissues (41). In female carriers of the same allele, reduced levels of sACE2, but higher levels of Ang (1–7) were previously detected (5), indicating an increase in mACE2/sACE2 ratio that is probably due to impediment in either ADAM17 docking on mACE2 cleavage site, or its efficacy in ectodomain shedding (29). In contrast to several earlier reports of rs2106809 C/C genotype connection with higher risk of hospitalization and ICU admission (42, 43), we failed to observe any significant association between this polymorphism and a severe form of COVID-19. However, our results suggest the presence of variant rs2106809 C allele as a prominent protective factor for in-hospital death due to COVID-19, but only among females. While this finding corresponds well to the expected genotype-related change in mACE2 and sACE2 levels, as well as to its inverse association with COVID-19 mortality, question arises why it applies to female population only. One of the plausible explanations we propose considers the location of *ACE2* gene on the X chromosome (28) within a region of incomplete X-chromosome inactivation (XCI) (44). Namely, for most of the X-linked genes, one allele is normally inactivated to balance its expression between females and males (45). However, some of them, including *ACE2* (44), escape XCI, resulting in female-biased *ACE2* gene expression (46), and thus a more pronounced effect of any functional *ACE2* genetic polymorphism, including rs2106809, in females. Finally, it should be noted that in our study *ACE2* rs2106809 deviates from HWE. Although HW disequilibrium in genetic studies could indicate issues like selection bias, population stratification, or genotyping errors (47), we assume the departure we observed is due to location of *ACE2* on the X chromosome, as it causes violation of one of the basic assumptions that HW principle relies on—that the locus should be autosomal (48).

*TMPRSS2* gene is located on chromosome 21 and encodes transmembrane protease TMPRSS2 (7). This enzyme represents one of the key factors for penetration of various viruses, including SARS-CoV-2, into the cells: upon binding to the ACE2 receptor, TMPRSS2 initiates fusion machinery between the viral particle and cell phospholipid membranes, and delivers the virion into the cytoplasm (7). TMPRSS2 is expressed in many human organs and tissues (49–51), and in most it is colocalized with ACE2, making them more susceptible targets for SARS-CoV-2 (52). Previous observations of more severe forms of COVID-19 in men as compared to women (53), as well as of decreased risk of severe respiratory illness in SARS-CoV-2-infected infants and children (54), led to speculations about the critical influence of well-known androgen-dependent upregulation of TMPRSS2 expression (55) on the clinical course of this disease. However, TMPRSS2 expression is regulated in organ- and tissue-specific manner (56), with inducing effect of male hormones being mainly responsible for prostate localization (14). In lungs, on the other hand, TMPRSS2 displays less prominent androgen responsiveness (56), and gene expression to a significant extent depends on the presence of genetic polymorphisms (10, 57).

*TMPRSS2* rs2070788 represents a regulatory intron variant, whose ancestral G allele leads to higher gene expression in lungs (57, 58). In line with the role of TMPRSS2 in SARS-CoV-2 infection, rs2070788 G allele has been repeatedly (10, 59, 60), although not consistently (61), associated with severe clinical presentation and increased mortality of several respiratory infectious diseases, including COVID-19. In our study, homozygous carriers of variant A allele were less likely to develop severe clinical presentation after SARS-CoV-2 infection. This finding corresponds well to the expected genotype effect, as well as to most of the previous studies investigating the risk of severe COVID-19 in the presence of variant rs2070788 allele. However, after controlling for other factors of influence, the genotype–phenotype association we observed remained significant only among females. Similar reports in the literature are not many, but the protective role of rs2070788 A/A genotype in female COVID-19 patients has been described previously (62). We believe the observed sex-related difference has a hormonal background: genetically determined decrease in TMPRSS2 levels appears more evident in females as compared to males, because females lack androgen-dependent upregulation of *TMPRSS2* gene expression.

There are several limitations in our study. Firstly, it was designed as a single centre study, which included only one population of respondents. Furthermore, our relatively small sample size could have reduced statistical power, while other potentially functional *ACE2* and *TMPRSS2* SNPs, which we did not genotype for, could have affected our results. Most importantly, we could not collect data on confounding variables, such as level of viral exposure, viral load, patient health habits, environmental influences, and the use of other drugs, which could have influenced the outcome.

## 5 Conclusion

In conclusion, our study highlights association between the presence of *TMPRSS2* rs2070788 and *ACE2* rs2106809 polymorphisms, and the severity and outcome of COVID-19, respectively, among female patients. These genetic variations may serve as important markers for identifying individuals at higher risk for severe disease and unfavorable outcomes, potentially guiding personalized treatment approaches in the future.

## Data Availability

The raw data supporting the conclusions of this article will be made available by the authors, without undue reservation.
