# Supplementary material for: ACE2 and TMPRSS2 genetic polymorphisms as potential predictors of COVID−19 severity and outcome in females
Source: Front Med (Lausanne). 2024 Dec 18;11:1493815. doi: 10.3389/fmed.2024.1493815 (PMC11688283; doi:10.3389/fmed.2024.1493815)
Supplement: Supplementary file 1 [file Table_1.DOCX]

Supplementary Table 1. *ACE2* allele and genotype frequency in COVID-19 cohort (n=178**). stratified by sex

|  | |  | **Frequency** | **[95% CI]** |
| --- | --- | --- | --- | --- |
| **Alleles** | |  |  |  |
| F: | rs2106809 C | | 12.5 (18/144) | [8.0; 19.0] |
|  | rs2074192 A | | 48.6 (70/144) | [40.6; 56.7] |
| M: | rs2106809 C | | 24.5 (26/106) | [19.6; 31.3] |
|  | rs2074192 A | | 34.0 (33/97) | [28.0; 41.3] |
| **Genotype groups*** | |  |  |  |
|  | **Additive model** | |  |  |
|  | rs2106809 | T/T | 79.2 (57/72) | [68.3; 87] |
|  |  | T/C | 16.7 (12/72) | [9.7; 27.1] |
|  |  | C/C | 4.2 (3/72) | [1.0; 12.2] |
|  | rs2074192 | G/G | 27.8 (20/72) | [18.8; 39.1] |
|  |  | G/A | 47.2 (34/72) | [36.1; 58.6] |
|  |  | A/A | 25.0 (18/72) | [16.4; 36.2] |
|  | **Dominant model** | |  |  |
|  | rs2106809 | T/T | 79.2 (57/72) | [68.3; 87.0] |
|  |  | T/C + C/C | 20.8 (15/72) | [13.0; 31.7] |
|  | rs2074192 | G/G | 27.8 (20/72) | [18.8; 39.1] |
|  |  | G/A + A/A | 72.2 (52/72) | [60.9; 81.2] |
|  | **Recessive model** | |  |  |
|  | rs2106809 | T/T + T/C | 95.8 (69/72) | [87.8; 99.0] |
|  |  | C/C | 4.2 (3/72) | [1.0; 12.2] |
|  | rs2074192 | G/G + G/A | 75.0 (54/72) | [63.8; 83.6] |
|  |  | A/A | 25.0 (18/72) | [16.4; 36.2] |

F – female; M – male; *female only; 95% CI – 95% confidence interval

**- genotyping for rs2074192 was successful in 168 patients only
