# Supplementary material for: ACE2 and TMPRSS2 genetic polymorphisms as potential predictors of COVID−19 severity and outcome in females
Source: Front Med (Lausanne). 2024 Dec 18;11:1493815. doi: 10.3389/fmed.2024.1493815 (PMC11688283; doi:10.3389/fmed.2024.1493815)
Supplement: Supplementary file 2 [file Table_2.DOCX]

Supplementary Table 2. TMPRSS2 alleles and genotype frequency in COVID-19 cohort stratified by sex

|  | |  | **Females** | | **Males** | |
| --- | --- | --- | --- | --- | --- | --- |
|  | |  | **Frequency** | **[95% CI]** | **Frequency** | **[95% CI]** |
| **Alleles** | |  |  |  |  |  |
|  | rs2070788 G | | 41.7 (60/144) | [33.9; 49.8] | 48.6 (103/212) | [41.9; 55.3] |
|  | rs4818239 C | | 45.3 (65/144) | [37.3; 53.3] | 48.1 (102/212) | [41.5; 54.8] |
| **Genotype groups** | | |  |  |  |  |
|  | **Additive model** | |  |  |  |  |
|  | rs2070788 | G/G | 18.1 (13/72) | [10.8;28.7] | 23.6 (25/106) | [16.5; 32.6] |
|  |  | G/A | 47.2 (34/72) | [36.1; 58.6] | 50.0 (53/106) | [40.7; 59.3] |
|  |  | A/A | 34.7 (25/72) | [24.8; 46.3] | 26.4 (28/106) | [18.9; 35.6] |
|  | rs4818239 | T/T | 31.9 (23/72) | [22.3; 43.5] | 27.4 (29/106) | [19.8; 36.6] |
|  |  | T/C | 45.8 (33/72) | [34.8; 57.3] | 49.1 (52/106) | [39.7; 58.4] |
|  |  | C/C | 22.2 (16/72) | [14.1; 33.2] | 23.6 (25/106) | [16.5; 32.6] |
|  | **Dominant model** | |  |  |  |  |
|  | rs2070788 | G/G | 18.1 (13/72) | [10.8; 28.7] | 23.6 (25/106) | [16.5; 32.6] |
|  |  | G/A+A/A | 81.9 (59/72) | [71.3; 89.2] | 76.4 (81/106) | [67.4; 83.5] |
|  | rs4818239 | T/T | 31.9 (23/72) | [22.3; 43.5] | 27.4 (29/106) | [19.8; 36.6] |
|  |  | T/C+C/C | 68.1 (49/72) | [56.5; 77.7] | 72.6 (77/106) | [63.4; 80.2] |
|  | **Recessive model** | |  |  |  |  |
|  | rs2070788 | G/G+GA | 65.3 (47/72) | [53.7; 75.2] | 73.6 (78/106) | [64.4; 81.1] |
|  |  | A/A | 34.7 (25/72) | [24.8; 46.3] | 26.4 (28/106) | [18.9; 35.6] |
|  | rs4818239 | T/T+T/C | 79.2 (57/72) | [68.3; 87.0] | 75.5 (80/106) | [66.4; 82.7] |
|  |  | C/C | 20.8 (15/72) | [13.0; 31.7] | 24.5 (26/106) | [17.3; 33.6] |

95% CI – 95% confidence interval
