# Supplementary material for: ACE2 and TMPRSS2 genetic polymorphisms as potential predictors of COVID−19 severity and outcome in females
Source: Front Med (Lausanne). 2024 Dec 18;11:1493815. doi: 10.3389/fmed.2024.1493815 (PMC11688283; doi:10.3389/fmed.2024.1493815)
Supplement: Supplementary file 3 [file Table_3.DOCX]

Supplementary Table 3. Summary of variable estimates from the best fitting models of multiple logistic regression analysis regarding severity and outcome of SARS-CoV-2 infection in males

|  | **Variables** | **B** | **S.E.** | **Wald** | ***p*** | **OR** | **[95%CI]** |
| --- | --- | --- | --- | --- | --- | --- | --- |
| **Severity** | LDH | 0.012 | 0.003 | 18.708 | **<0.001** | 1.012 | [1.007; 1.017] |
|  | *TMPRSS2* rs4818239^a^ | -1.680 | 0.944 | 3.166 | 0.075 | 0.186 | [0.029; 1.186] |
|  | Constant | -6.965 | 1.520 | 21.002 | 0.000 | 0.001 |  |
| **Outcome** | N/L | 0.163 | 0.065 | 6.294 | 0.012 | 1.177 | [1.036; 1.337] |
|  | LDH | 0.004 | 0.002 | 5.668 | 0.017 | 1.004 | [1.001; 1.008] |
|  | ACE2 rs2106809^b^ | -1.362 | 1.225 | 1.235 | 0.266 | 0.256 | [0.023; 2829] |
|  | Constant | -6.722 | 1.724 | 15.206 | 0.000 | 0.001 |  |

Values in bold indicate statistically significant results. LDH – lactat dehydrogenase; N/L – neutrophil to lymphocyte ratio; B – the regression coefficient; S.E. – the standard error; Wald χ2– Wald test statistics for the degree of freedom of 1 (df=1); OR – odds ratio; 95% CI – the 95% confidence interval for the estimated OR; *p* – the probability

^a^ – recessive model. G/G + G/A as reference category

^b^ – dominant model. A/A as reference category
